# Using the hybrid EMD-BPNN model to predict the incidence of HIV in Dalian, Liaoning Province, China, 2004–2018

**DOI:** 10.1186/s12879-022-07061-7

**Published:** 2022-01-29

**Authors:** Qingyu An, Jun Wu, Jun Meng, Zhijie Zhao, Jin Jian Bai, Xiaofeng Li

**Affiliations:** 1 Dalian Center for Disease Control and Prevention, Dalian, 116021 Liaoning China; 2 School of Public Health, Dalian Medical University, Dalian, 116044 Liaoning China

**Keywords:** HIV, EMD, BPNN, ARIMA

## Abstract

**Background:**

Acquired immunodeficiency syndrome (AIDS) is a malignant infectious disease with high mortality caused by HIV (human immunodeficiency virus, and up to now there are no curable drugs or effective vaccines. In order to understand AIDS’s development trend, we establish hybrid EMD-BPNN (empirical modal decomposition and Back-propagation artificial neural network model) model to forecast new HIV infection in Dalian and to evaluate model’s performance.

**Methods:**

The monthly HIV data series are decomposed by EMD method, and then all decomposition results are used as training and testing data to establish BPNN model, namely BPNN was fitted to each IMF (intrinsic mode function) and residue separately, and the predicted value is the sum of the predicted values from the models. Meanwhile, using yearly HIV data to established ARIMA and using monthly HIV data to established BPNN, and SARIMA (seasonal autoregressive integrated moving average) model to compare the predictive ability with EMD-BPNN model.

**Results:**

From 2004 to 2017, 3310 cases of HIV were reported in Dalian, including 101 fatal cases. The monthly HIV data series are decomposed into four relatively stable IMFs and one residue item by EMD, and the residue item showed that the incidence of HIV increases firstly after declining. The mean absolute percentage error value for the EMD-BPNN, BPNN, SARIMA (1,1,2) (0,1,1)_12_ in 2018 is 7.80%, 10.79%, 9.48% respectively, and the mean absolute percentage error value for the ARIMA (3,1,0) model in 2017 and 2018 is 8.91%.

**Conclusions:**

The EMD-BPNN model was effective and reliable in predicting the incidence of HIV for annual incidence, and the results could furnish a scientific reference for policy makers and health agencies in Dalian.

**Supplementary Information:**

The online version contains supplementary material available at 10.1186/s12879-022-07061-7.

## Background

The full name of AIDS is acquired immunodeficiency syndrome, which is a malignant infectious disease with high mortality caused by human immunodeficiency virus infection [1], and has become one of global public health problem [2]. Since the first case reported in China [3], there has been a tendency toward an increase in HIV epidemics in the country [4]. As of September 30 in 2013, a total of 434,000 HIV infections and AIDS patients were reported in China, and the main way of transmission in China was transsexual transmission at present [5]. Up to now, there are no curable drugs or effective vaccines. Therefore, establish forecasting model of HIV, so as to find the development trend of HIV, is of great significance to the HIV prevention and control work. For the epidemic trend of HIV, its influencing factors are complex, including population, economy, behavior and environment. At present, China has not fully carried out the monitoring and collection of data on HIV-related influencing factors, so it is difficult to establish a prediction model of HIV by analyzing the influencing factors. In recent years, some scholars have used ARIMA model, GM (1,1) model, BP neural network model to predict the incidence trend of HIV. For example, Liang et al. [6] using G (1, 1) modeling method to fit the incidence of HIV in Jiangsu province, the relative error was 23.89%. GM is a mathematical model based on grey system theory, which can systematically predict the trend of variable change. GM (1,1) is the simplest form of model, the basic steps of establishing the model are firstly cumulate the irregular original data to a regular sequence of data and then build the differential equation, so as to predict the future development trend of the disease [7]. Yang et al. [8] using ARIMA to build modeling HIV incidence from 2000 to 2014 in China and the mean absolute percentage error was 19.90%. Wu et al. [9] use Back-propagation neural network (BP-ANN) as a model to predict HIV prevalence, and the ratios of accuracy for training, calibration and detection were 93.94%, 88.48% and 89.60%, respectively.

In order to improve the accuracy of the prediction model, in this study, we took the HIV incidence data from 2004 to 2018 as an example, established a two-stage EMD (empirical mode decomposition)-BPNN (back-propagation artificial neural network model) model to forecast HIV in Dalian and the prediction results will provide the basis for AIDS monitoring and prevention in Dalian.

## Materials and methods

### Study area

Dalian is the main coastal city of Liaoning Province, China and a major tourist city located at 38° 43′–40° 10′ N latitude and 120° 58′–123° 31′ E longitude. It had a registered population of 5.949 million in 2017. Dalian has a warm continental monsoon climate and is in a marine temperate zone. The average annual temperature is 10 ℃ with a maximum of 35 ℃, and a minimum of − 28 ~ − 18 ℃. The average rainfall is 550–800 mm and the total hours of annual sunshine is 2500–2800 h [10].

### Data collection

The HIV was a notifiable monitoring communicable disease in China. The clinicians are required to report HIV cases through the China information system for disease control and prevention within 24 h. During 1995 to 2008, there was 320 cases of HIV infection reported in Dalian, and the average annual growth rate of incidence was 31.06% [11]. Yearly incidence rate of HIV during the period of 1999 to 2018 and monthly incidence number of HIV cases in Dalian during the period of 2004 to 2018 was obtained from above information system’s statistical report around the beginning of February in the next year. The diagnostic criteria of HIV is compliance with diagnostic criteria of HIV infection by Secretariat of the Professional Committee on Infectious Diseases Standards and Secretariat of the Professional Committee on Parasitic Disease Standards of the Ministry of Health of the People’s Republic of China in May, 2010 [12].

### Data analysis

The monthly HIV data series are decomposed by EMD method, and then all decomposition results are used as training and testing data to establish BPNN model, namely BPNN was fitted to each intrinsic mode function (IMF) and residue separately, the predicted value is the sum of the predicted values from the models, and the annual incidence equal to the sum of the predicted results of the 12 months. Meanwhile, using monthly HIV data to established BPNN and SARIMA model and using yearly HIV incidence rate to established ARIMA model to compare the predictive ability with EMD-BPNN model.

### Empirical modal decomposition

Empirical modal decomposition is a self-adaptive decomposition method which is developed for non-stationary and nonlinear signal processing, was proposed by Huang et al. [13]. After EMD processing, complex signals can be decomposed into several intrinsic mode function (IMF) components based on the local characteristic time scale of the signal from high to low frequency and a residue.

The expression of the model is:

$$s(t) = \sum\limits_{t = 1}^{n} {imfi} (t) + rn(t)$$

where $s(t)$ denote original signals, $imfi(t)$ denote the $i$th intrinsic mode function, and $i = 1,2, \cdots ,n$,$rn(t)$ denote residual signal.

The IMF must meet the following two conditions:

1. In the whole signal data series, the number of extrema points must be equal to the number of Zero or differ by one at most;
2. The mean value of envelope defined by maximum and minimum must be equal and zero [14].

Empirical modal decomposition of monthly incidence data of HIV from 2004 to 2017 was performed by using MATLAB 2014.

### BP artificial neural network

**Fig. 1:**
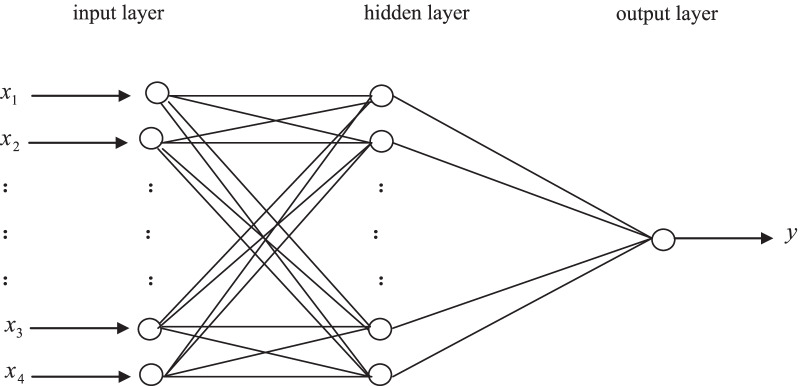
The topological structure of BPNN model

A back-propagation artificial neural network is a multi-layer feed-forward neural network trained by error back propagation algorithm [15]. Its topological structure consists of three layers, namely input layer, hidden layer and output layer, each of which consists of some joints. The hidden layer connects input and output layer, and their correlations are reflected by relevant coefficients, namely connection weights. Usually, through self-learning or self-training of artificial neural network, repeatedly adjust these coefficients until reaching a goal of well-trained model (Fig. 1).

The learning process of BP algorithm consists of two processes: forward propagation and backward propagation. In the forward propagation process, the input information is transferred from the input layer to the output layer through the hidden layer. If the output layer does not get the desired output, it is transferred to the back propagation. The error signal is returned along the original connection path, and the connection weights between joint in each layer are modified, so the network parameters are adjusted repeatedly to make the minimized error function [16].

BPNN model was performed by using DPS data processing system.

### The ARIMA and SARIMA model

A SARIMA model can be described as ARIMA (p, d, q) multiplied by (P, D, Q), where the terms p, d, q represent ordinary components, while P, D, Q represent seasonal components [17]. Simulated the monthly incidence data of HIV from 2004 to 2017 and the yearly incidence rate of HIV from 1999 to 2016 with the Box-Jenkins modeling approach, we fitted the ARIMA model and the SARIMA model to HIV incidence, and then used the fitted model to out-of-sample predict yearly incidence rate of HIV for the year 2017 and 2018 and monthly incidence of HIV for the year 2018, respectively.

Firstly, autocorrelation function (ACF) graph and partial autocorrelation function (PACF) graph were used to identify the possible values for the autoregressive or moving average components. Secondly, estimates of the model’s parameters were obtained by the least squares method according to the different values of p, d, q and p, d, q, P, D, Q. Thirdly, compared the models by the Akaike information criterion (AIC), where the preferred model was the one with the lowest AIC value. Finally the goodness of fit of each model was verified by plotting the autocorrelation and partial autocorrelation of residuals and by using the Ljung-Box test.

SARIMA and ARIMA model was performed by using 11.5 version of Statistical Package for Social Sciences (SPSS11.5) with a significant level of p < 0.05.

### Performance comparison

We used ARIMA, BPNN and the seasonal ARIMA model as single time series prediction method to predict yearly and monthly incidence rate of HIV and compare the predictive ability with EMD-BPNN model.

The criterion for comparing the predictive ability of the models was the absolute percentage error defined as:

$$e = \left| {\frac{{(xt - \hat{x}t)}}{xt}} \right| \times 100\% ,$$

where $xt$ and $\hat{x}t$ denote observed and fitted value at time point respectively. Thus, the preferred model is the one with the lowest mean absolute percentage error.

## Results

### Descriptive analysis

From 2004 to 2017, 3310 cases of HIV were reported (3122 males and 188 females) in Dalian, Liaoning Province, China, including 101 fatal cases. The average incidence rate is 4.01/100,000 population, the average mortality rate is 0.12/100,000 population, and the case fatality rate is 3.05%. The incidence of the HIV is mainly concentrated in the age group of 20 to 49 years old which has 2751 cases of HIV reported and coming to 83.11% in total.

### Decomposition results of the original monthly HIV data series by EMD

**Fig. 2:**
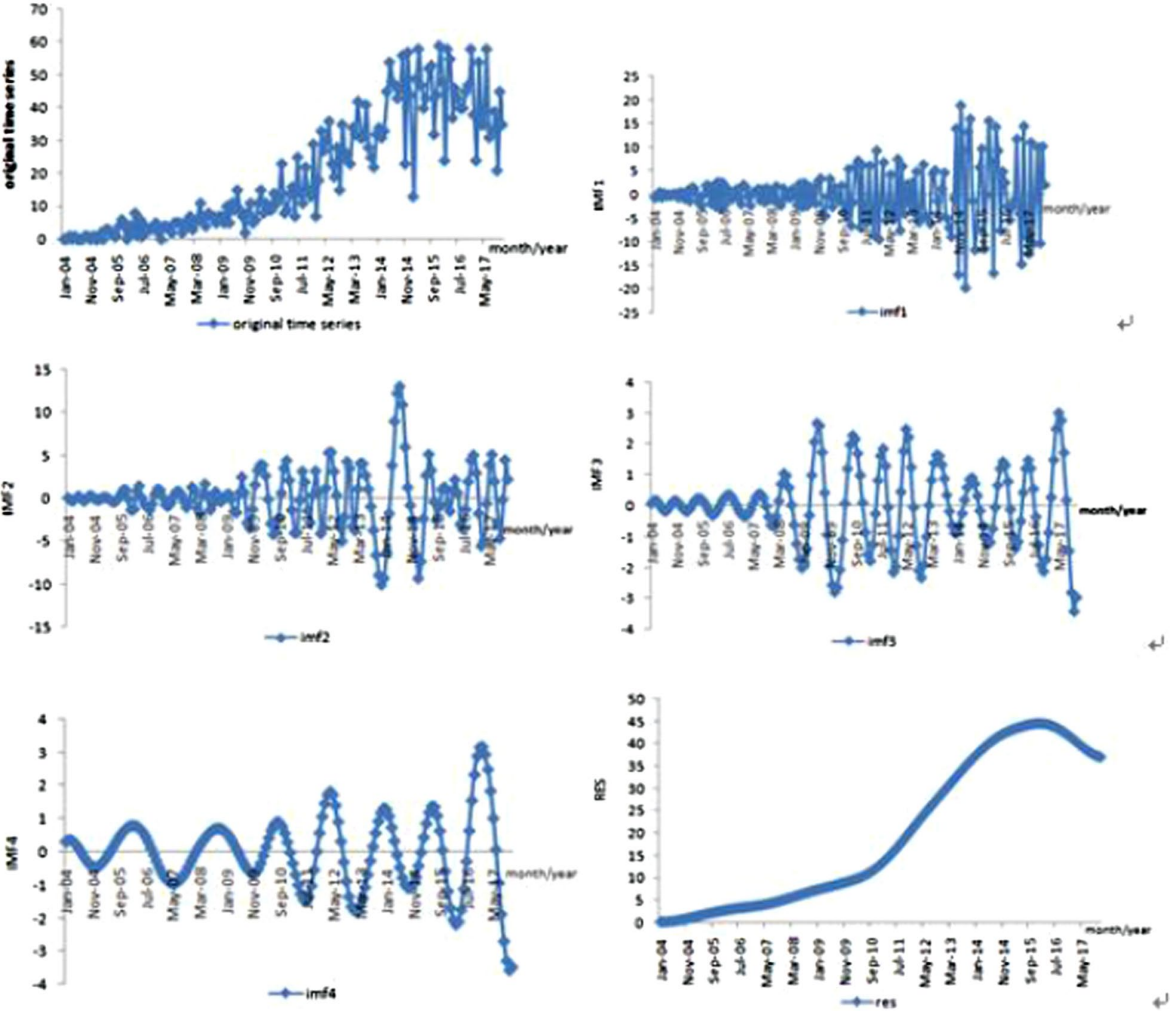
Decomposition results of the original monthly HIV data series in Dalian by EMD

The original monthly HIV data series from 2004 to 2017 was decomposed by EMD. Four independent IMFs and one residue item were obtained. Figure 2 showed decomposition results of the original monthly HIV data series of Dalian by EMD. As shown in figure, IMF presents the oscillation characteristics in order from high frequency to low frequency. Specifically, IMF1, IMF2 and IMF3 represented high frequency variable and large fluctuation range, IMF4 represented low frequency component, and the fluctuation range was small. The residue item is the last one, which is reflect the overall trend of the original data series. In this study, the residue item showed that the monthly incidence data of HIV in Dalian during2004 to 2015 is increasing and during 2016 to 2017 is declining.

### Fitting and forecasting IMFs and residue by BPNN model

In this study, we establish a BPNN model to predict the decompositions IMF1 to IMF4 and the residue item. Because of the monthly data series, we took the every 12 IMF and residue item as the input, the thirteenth IMF and residue item as output. The parameters of model were as follows. We test 12 candidate the node number of one hidden layer from 1 to 12, and then, we calculated fitting residuals to determine the optimal value. Table 1 showed the node number of one hidden layer and the model’s fitting residuals. The node value was 12 had the smallest fitting residuals (0.003581%), so we choice the node number of one hidden layer was 12, the minimum training velocity was 0.1, the permissible error was 0.001, the maximum iteration number was 1000, and the values of input nodes were standardization transformation. After 1000 run, IMF1 to IMF4 fitting residuals were 0.003581%, 0.001022%, 0.000354% and 0.000218%, residue item fitting residuals was 0.00132%, and the model fitted well.

**Table 1:** The different node number of hidden layer and the model’s fitting residuals

The node number of one hidden layer	Fitting residuals (%)
12	0.003581
11	0.003717
10	0.00381
9	0.003985
8	0.00363
7	0.003737
6	0.004291
5	0.00458
4	0.006355
3	0.008446
2	0.010625
1	0.019218

Table 2 showed the number of predicted cases from January to December 2018 obtained from the EMD-BPNN model, and the annual incidence equal to the sum of the predicted results of the 12 months. The observed and predicted values in 2018 were relatively close to each other, and the absolute percentage error value for the model was 7.80%.

**Table 2:** The number of HIV cases observed during 2018 and predicted values obtained from the EMD-BPNN model

Month	IMF1	IMF2	IMF3	IMF4	Residue item	Predicted value	Observed value
1	− 0.4262	− 2.6947	− 2.1377	− 3.1996	38.7255	30.2673	28
2	− 1.2183	− 1.6887	− 1.0954	− 3.1254	39.1768	32.049	30
3	5.6477	3.4268	0.1188	− 3.193	39.5275	45.5278	46
4	− 9.2236	4.842	1.3709	− 3.3332	39.9215	33.5776	50
5	9.1589	2.0379	2.3587	− 3.4737	40.2396	50.3214	66
6	− 5.1057	− 1.0484	2.5335	− 3.5432	40.5752	33.4114	52
7	0.0473	− 0.8585	2.2695	− 3.5595	40.9097	38.8085	45
8	2.6705	0.1333	1.4116	− 3.5628	41.104	41.7566	41
9	2.1908	0.5143	0.0681	− 3.5633	41.2913	40.5012	38
10	− 9.0812	0.9662	− 1.0834	− 3.5633	41.4653	28.7036	37
11	7.1544	0.6492	− 2.2042	− 3.5633	41.5809	43.617	25
12	1.4365	− 0.7369	− 2.798	− 3.5633	41.6835	36.0218	35
Total						454.563	493
Absolute percentage error (%)							7.797

### Performance comparison analysis

To understand the performance of the hybrid EMD-BPNN model, the predicted results of the hybrid EMD-BPNN model were compared with BPNN, the seasonal ARIMA and ARIMA.

#### BPNN

Because of the monthly data series, we took the every 12 month incidences as the input, the thirteenth month incidence as output. The parameters of model were as follows. The node number of one hidden layer was 12, the minimum training velocity was 0.1, the permissible error was 0.001, the maximum iteration number was 1000, and the values of input nodes were standardization transformation. After 1000 run, fitting residuals were 0.002840%, and the model fitted well.

Table 3 showed the number of predicted cases from January to December 2018 obtained from the BPNN model, and the annual incidence equal to the sum of the predicted results of the 12 months. The absolute percentage error for the model was 10.79%.

**Table 3:** The number of HIV cases observed during 2018 and predicted values obtained from the BPNN model

Month	Predicted value	Observed value
1	42.2638	28
2	48.471	30
3	37.5714	46
4	42.3757	50
5	56.0465	66
6	47.1118	52
7	52.4077	45
8	43.8192	41
9	48.7503	38
10	49.2205	37
11	30.0177	25
12	48.1137	35
Total	546.1693	493
Absolute percentage error (%)		10.785

#### SARIMA

**Fig. 3:**
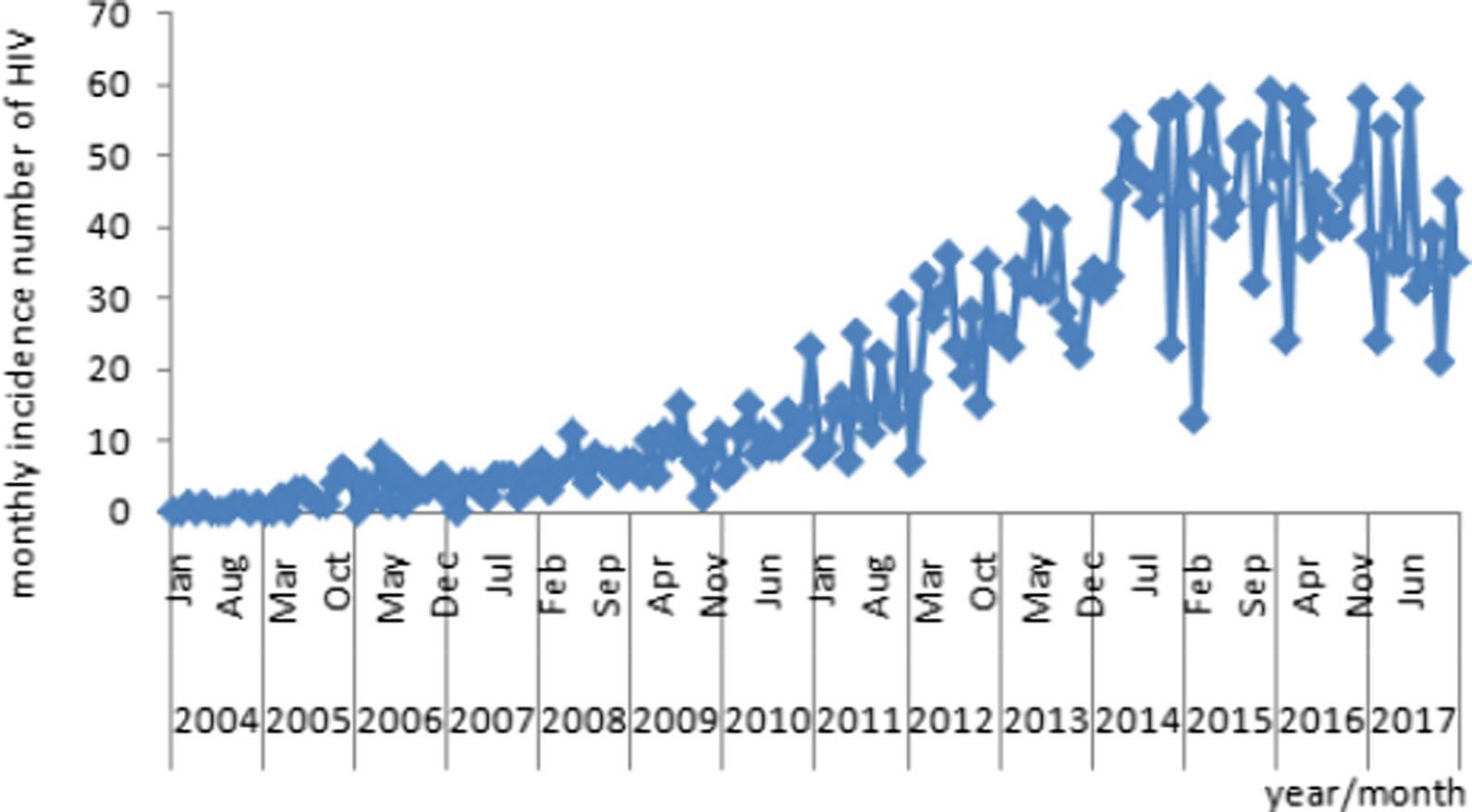
The sequence of HIV incidence from 2004 to 2017

Firstly, the sequence of HIV incidence from 2004 to 2017 was used to determine the stability of the sequence. According to Fig. 3, the series show non-stationary mean, so it is necessary to stabilize the variance of HIV incidence by computing its natural logarithm. The monthly data used in this study, so the time series was differenced once at the seasonal level and non-seasonal level. The transformed HIV incidence showed far less dispersion and become stationary because of that the number of HIV cases fluctuated around the mean. All further statistical procedures were performed on the transformed HIV incidence.

Secondly, because of the time series after one seasonal difference and non-seasonal difference, d equals one and D equals one. The slow decay in the ACF at lags 1–2 (autocorrelation1 = − 0.578, autocorrelation2 = 0.222) suggests that non-seasonal q equals to one or two, while also it may be zero, and a PACF cutoff at lag 1 (partial autocorrelation1 = − 0.578) suggests that non-seasonal p equals one. The slow decay in the ACF at lags 11–13 (autocorrelation11 = 0.315, autocorrelation12 = − 0.408, autocorrelation13 = 0.206), and a PACF cutoff at lag 11 (partial autocorrelation11 = 0.298) suggests seasonal Q equals zero, one or two and seasonal P equals zero.

The model parameters for SARIMA (1,1,2) (0,1,1)_12_ model, the non-seasonal autoregressive parameters is estimated as − 0.839 (SE = 0.142, t = − 5.925, p = 0.000), non-seasonal moving average parameters one is estimated as 0.183 (SE = 0.176, t = 1.036, p = 0.302), non-seasonal moving average parameters two is estimated as 0.614 (SE = 0.168, t = 3.649, p = 0.000) and seasonal moving average parameter is 0.952 (SE = 0.267, t = 3.567, p = 0.000) respectively.

Table 4 showed AIC values for the SARIMA models corresponding to different choices of p, q, P and Q. The SARIMA (1,1,2) (0,1,1)_12_ model has the lowest AIC.

**Table 4:** Residual diagnostics for different SARIMA models

Model	Standard error (SE)	Log-likelihood	(AIC)	Schwarz Bayesian criterion (BIC)
ARIMA (1, 1, 0) (0, 1, 0) _12_	0.612	− 133.790	271.579	277.505
ARIMA (1, 1, 0) (0, 1, 1) _12_	0.485	− 106.333	218.666	227.555
ARIMA (1, 1, 0) (0, 1, 2) _12_	0.462	− 105.583	219.166	231.017
ARIMA (1, 1, 1) (0, 1, 0) _12_	0.532	− 113.014	232.028	240.916
ARIMA (1, 1, 1) (0, 1, 1) _12_	0.402	− 86.042	180.085	191.936
ARIMA (1, 1, 1) (0, 1, 2) _12_	0.424	− 86.563	183.125	197.939
ARIMA (1, 1, 2) (0, 1, 0) _12_	0.521	− 109.498	226.997	238.848
ARIMA (1, 1, 2) (0, 1, 1) _12_	0.401	− 83.908	177.816	192.630
ARIMA (1, 1, 2) (0, 1, 2) _12_	0.403	− 83.842	179.685	197.462

**Fig. 4:**
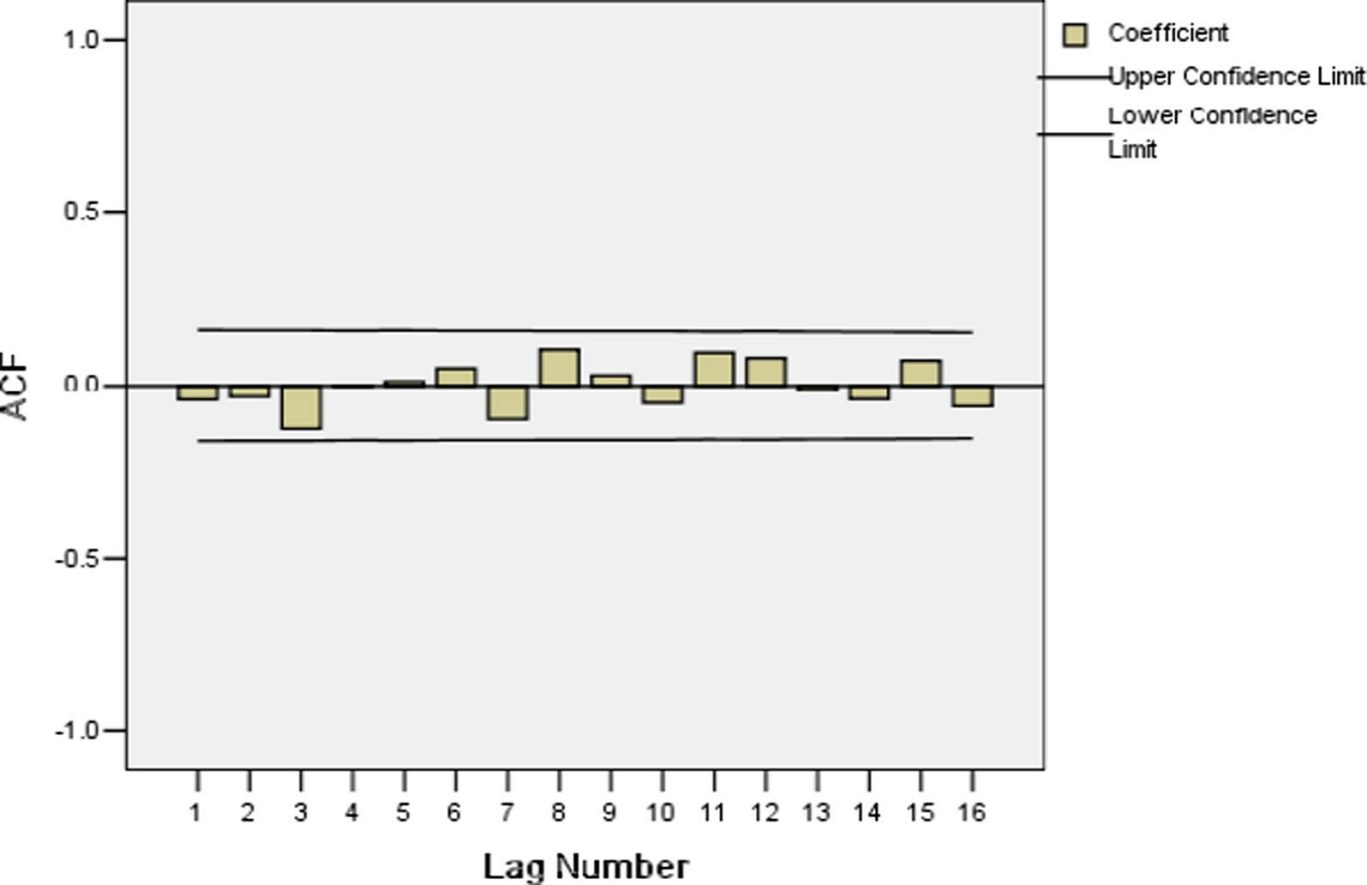
Autocorrelation function (ACF) of residuals for SARIMA (1,1,2)( 0,1,1)_12_ model

**Fig. 5:**
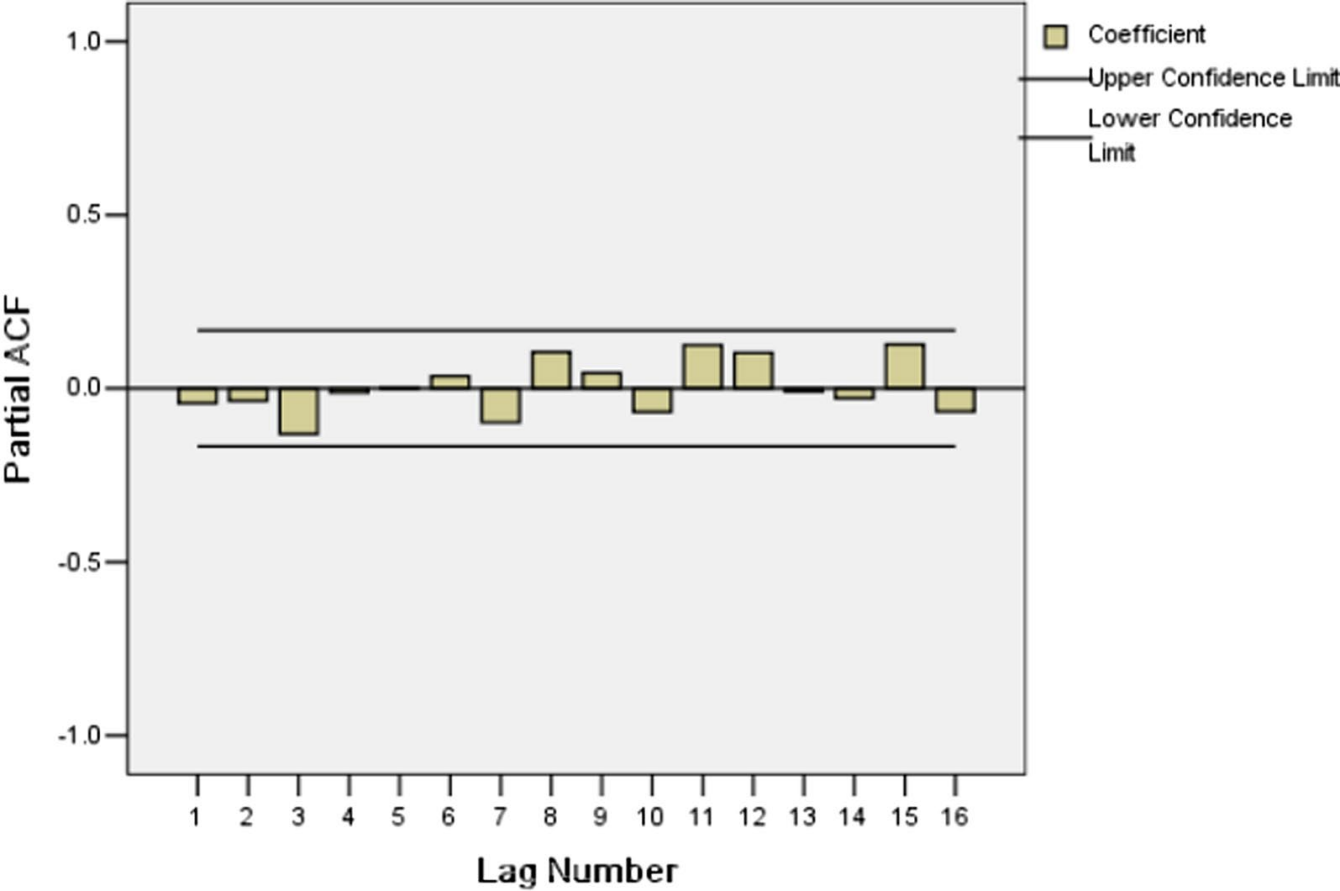
Partial autocorrelation functions of (PACF) of residuals for SARIMA ((1,1,2) (0,1,1)_12_

The Ljung-Box statistic of ARIMA (1, 1, 2) (0, 1, 1) _12_ model showed that there were high p-values associated with the statistics. The null hypothesis of independence in this residual time series cannot be rejected (Table 5). The plots of the ACF and PACF of the residuals show no remaining temporal correlation (Figs. 4, 5). Thus it can be concluded that the ARIMA (1, 1, 2) (0, 1, 1) _12_ model identified fit the data well.

**Table 5:** Autocorrelations analysis results of residuals for SARIMA (1,1,2)( 0,1,1)_12_ model

LAG	Autocorrelation	Standard error	Box-Ljung Statistic		
			Value	Degrees of freedom	P value
1	− 0.043	0.081	0.276	1.000	0.599
2	− 0.034	0.081	0.454	2.000	0.797
3	− 0.127	0.081	2.927	3.000	0.403
4	0.002	0.080	2.927	4.000	0.570
5	0.013	0.081	2.953	5.000	0.707
6	0.051	0.080	3.360	6.000	0.762
7	− 0.099	0.080	4.902	7.000	0.672
8	0.106	0.080	6.655	8.000	0.574
9	0.031	0.080	6.803	9.000	0.658
10	− 0.052	0.080	7.236	10.000	0.703
11	0.097	0.079	8.733	11.000	0.647
12	0.081	0.079	9.778	12.000	0.635
13	− 0.013	0.079	9.805	13.000	0.710
14	− 0.041	0.079	10.077	14.000	0.757
15	0.074	0.078	10.974	15.000	0.754
16	− 0.062	0.078	11.608	16.000	0.771

Table 6 showed out-of-sample predicted values obtained from the SARIMA (1,1,2) (0,1,1)_12_ model and the results were compared with the observed number of HIV cases in 2018. The absolute percentage error value for the model was 9.48%.

**Table 6:** Observed number of HIV cases in 2018 and predicted values obtained from SARIMA (1,1,2)(0,1,1)_12_ model

Month	Observed value	Predicted value	Predicted value95%CI
1	28	33.128	14.607 ~ 75.130
2	30	24.133	10.639 ~ 54.742
3	46	45.609	19.774 ~ 105.198
4	50	43.043	18.563 ~ 99.809
5	66	39.427	16.782 ~ 92.624
6	52	43.186	18.297 ~ 101.933
7	45	35.672	14.906 ~ 85.371
8	41	35.021	14.572 ~ 84.166
9	38	37.461	15.431 ~ 90.938
10	37	28.312	11.602 ~ 69.090
11	25	37.745	15.270 ~ 93.297
12	35	43.519	17.519 ~ 108.106
Total	493	446.256	
Absolute percentage error (%)		9.482	

#### ARIMA

**Fig. 6:**
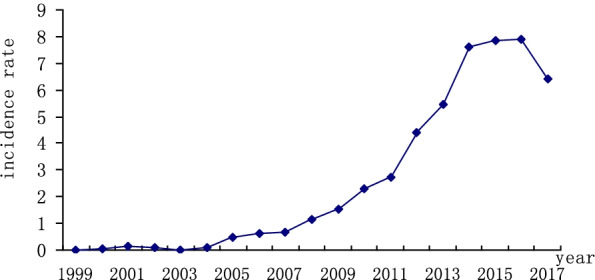
The sequence of HIV incidence rate from 1999 to 2016

**Fig. 7:**
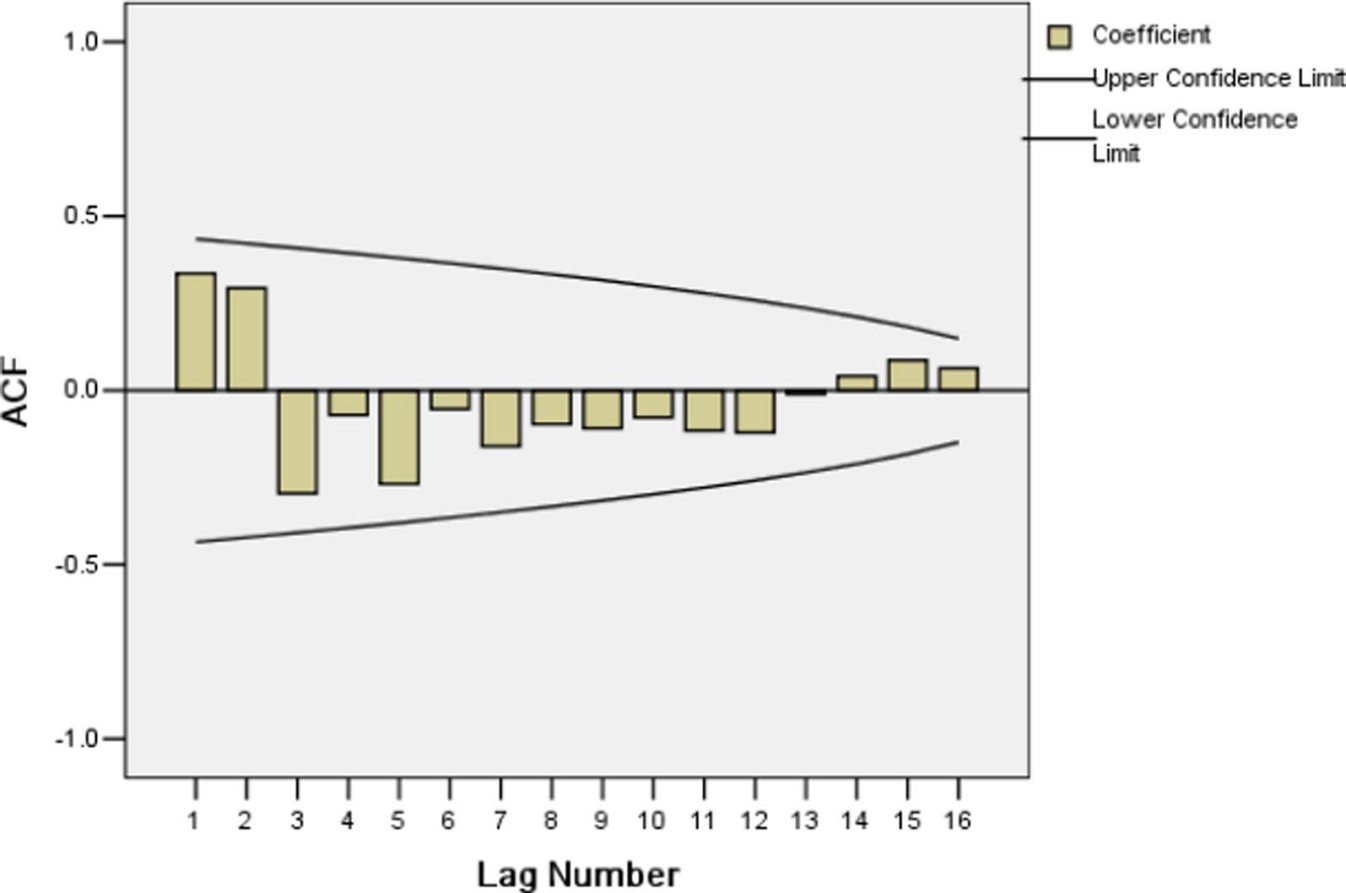
Autocorrelation function (ACF) for HIV incidence rate series with differenced once at the non-seasonal level

Firstly, the sequence of HIV incidence rate from 1999 to 2016 was used to determine the stability of the sequence. According to Fig. 6, the series show non-stationary mean, so the time series was differenced once at the non-seasonal level. The transformed HIV incidence become stationary because of that the incidence rate of HIV fluctuated around the mean (Fig. 7). All further statistical procedures were performed on the transformed HIV incidence.

**Fig. 8:**
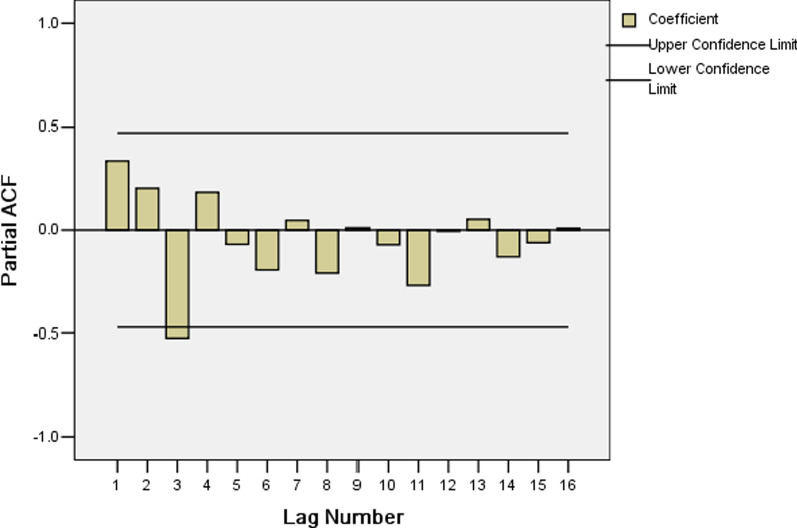
Partial autocorrelation functions of (PACF) for HIV incidence rate series with differenced once at the non-seasonal level

Secondly, because of the time series after one non-seasonal difference, d equals one. In the ACF, the coefficients are within the confidence interval (Fig. 7), suggests that non-seasonal q equals zero, and a PACF cutoff at lag 3 (partial autocorrelation1 = − 0.520) suggests that non-seasonal p equals three (Fig. 8).

The model parameters for ARIMA (3,1,0) model, the non-seasonal autoregressive parameters one is estimated as 0.603 (SE = 0.196, t = 3.078, p = 0.008), autoregressive parameters two is estimated as 0.533 (SE = 0.200, t = 2.663, p = 0.019), autoregressive parameters three is estimated as − 0.791 (SE = 0.204, t = − 3.882, p = 0.002) respectively.

The Ljung-Box statistic of ARIMA (3, 1, 0) model showed that there were high p-values associated with the statistics. The null hypothesis of independence in this residual time series can’t be rejected (Table 7). The plots of the ACF and PACF of the residuals show no remaining temporal correlation. Thus it can be concluded that the ARIMA (3,1,0) model identified fit the data well.

**Table 7:** Autocorrelations analysis results of residuals for ARIMA (3,1,0) model

LAG	Autocorrelation	Standard error	Box-Ljung Statistic		
			Value	Degrees of freedom	P value
1	0.135	0.217	0.384	1.000	0.536
2	0.084	0.211	0.543	2.000	0.762
3	− 0.199	0.204	1.489	3.000	0.685
4	0.015	0.197	1.494	4.000	0.828
5	− 0.180	0.190	2.395	5.000	0.792
6	− 0.041	0.183	2.446	6.000	0.875
7	− 0.147	0.175	3.150	7.000	0.871
8	− 0.129	0.167	3.749	8.000	0.879
9	0.030	0.158	3.785	9.000	0.925
10	− 0.009	0.149	3.788	10.000	0.956
11	− 0.110	0.139	4.415	11.000	0.956
12	− 0.114	0.129	5.193	12.000	0.951
13	0.039	0.118	5.302	13.000	0.968
14	− 0.009	0.105	5.309	14.000	0.981
15	0.067	0.091	5.848	15.000	0.982
16	0.054	0.075	6.365	16.000	0.984

The predict incidence rates of 2017 and 2018 obtained from the ARIMA (3,1,0) model were 5.569 per 100,000 population and 4.433 per 100,000 population respectively. Compared to the actual value of 5.266 per 100,000 population and 5.041 per 100,000 population, the average absolute percentage error for the model was 8.91%.

## Discussion

As one of the important public health problems in the world, AIDS has caused more and more serious harm to human health. It is one of the three global threats facing human beings, especially in developing countries [18]. According to a new report released by UNAIDS on the eve of World AIDS Day 2021, 1.5 million people were newly infected with HIV in 2020 [19]. The prevention and treatment of AIDS faces great challenges. From 2004 to 2017, 3310 cases of HIV were reported in Dalian, Liaoning Province, China, the average incidence rate is 4.01/100,000 population, the average mortality rate is 0.12/100,000 population, and the case fatality rate is 3.05%. The average incidence rate was lower than national level [20] and higher than the reported average incidence levels of some part of southern city in China [21, 22].

The prediction of infectious diseases can detect the trend of disease development in time. Therefore, if an area has continuous HIV surveillance data, the incidence number of HIV can be predicted by establishing mathematical models, and the results could be used to the HIV monitoring and provide the scientific basis for prevention strategies of the area. It makes AIDS prevention and control work more targeted, predictable and initiative. In this study, two stage EMD-BPNN model was established to predict the incidence trend of HIV in Dalian. Firstly, the monthly original incidence data of HIV from 2004 to 2017 was decomposed into a number of component and residue by empirical modal decomposition, then the component and residue items were taken as data sets to establish the neural network model. Two-stage EMD-BPNN advantage lied in compared with the prediction model established by direct application of original data, the complexity of the model can be effectively reduced. Because the disease incidence data often had the characteristics of non-linear and non-stationary, but the components obtained through empirical modal decomposition belonged to the variable set with the same frequency, and the residue term belonged to the increasing or decreasing variable, so it was easy to fit.

The other advantage of EMD is that can visually expressed the overall trend of the sequence. As seen from res curve in Fig. 2 during 2004 to 2015 the monthly incidence of HIV is increasing and during 2016 to 2017the monthly incidence of HIV is declining. This may be with the intensification of monitoring and surveillance in the period from 2004 to 2015, the number of newly discovered past infection increased and the number of new HIV-infected persons continued to increase. But with the implementation of preventive and control measures such as national policy, behavioral intervention, health education, et al., the number of new HIV infections declined since 2016. Although the monthly incidence of HIV dropped slightly in 2016 and 2017, but it did not show a significant downward trend. According to the analysis of the past HIV data in Dalian, sexual transmission was the major transmission route and an increase of prevalence was noticed among MSM [23]. So in order to prevent the AIDS epidemic, it is suggested that the health administration department continue to strengthen the efforts and scope for the high risk behavior intervention among MSM.

In this study, to further assess the prediction performance of the hybrid EMD-BPNN model, the absolute percentage error was utilized to measure performance. According to the comparison of EMD-BPNN, BPNN, SARIMA model for monthly HIV data series and ARIMA model for yearly HIV incidence rate series, the SARIMA model performed better than BPNN, but worse than EMD-BPNN model, and the ARIMA model performed slightly worse than EMD-BPNN model. In BPNN model, since the calculated data for the infection incidence prediction includes the incidence of infection in the previous period, the prediction effect for the long-term prediction result is poor due to the cumulative error. It is important to point out forecasting model influenced by outbreak for instance COVID-19. According to the results of a study on the impact of COVID-19 on HIV case reporting in China, reports of HIV cases have been significantly affected by COVID-19. Specifically, the number of reported HIV cases has significantly decreased during COVID-19 outbreak [24]. In addition, the proportion of HIV cases reported during the COVID-19 outbreak that were detected late was higher than in the other two non-COVID-19 outbreak years. In other words, due to the COVID-19, the original pattern of HIV has changed significantly. But when we do infectious disease prediction, we extrapolate it based on the principle of inertia of the data, that is, in the time series the diseases need to be in the same or similar natural state, so if the HIV incidence data of COVID-19 outbreak years and non-outbreak years are mixed together as basic data for prediction, it is bound to occur large errors and even impossible to conduct model. Therefore, in order to understand the trend of HIV in natural state, how to correct the HIV incidence data in the year of COVID-19 outbreak will become an important topic for further study. At the same time, as the incidence of HIV is affected by many natural and social factors, the established prediction model is not immutable. In future research, appropriate prediction model should be re-established according to the Additional file 1.

This study had several limitations. First, there might be many factors affecting HIV incidence, such as population, economy, behavior and environment, which can all contribute to and interact in the HIV transmission cycle, however, the availability of these data is limited. Second, the data of HIV incidence was obtained from passive surveillance, resulting in a potential underreporting of HIV cases, influencing the precision of our analysis.

## Conclusions

In brief, the original monthly HIV data series were decomposed into four relatively stable IMFs and one residue item using the EMD method, and then all decomposition results are used as training and testing data to establish the BPNN model, the final prediction of monthly HIV data equal to the sum of the predicted results of the each decomposition, and the annual incidence equal to the sum of the predicted results of the 12 months. Compared with BPNN, SARIMA for monthly HIV data series and ARIMA model for yearly HIV incidence rate series, the hybrid EMD-BPNN model has performs better than single times series prediction model like above three models, namely the hybrid model improve the prediction accuracy. Therefore, it can be considered that the hybrid EMD-BPNN model was effective and reliable in predicting the incidence of HIV in Dalian and the results could furnish a scientific reference for policy makers and health agencies.

## Supplementary Information

**Additional file 1:** Monthly cases of HIV in Dalian, Liaoning province, China, during 2019 to 2020.

## Data Availability

All data generated or analysed during this study are included in this published article and its additional information files.

## Abbreviations

AIDS: Acquired immunodeficiency syndrome
HIV: Human immunodeficiency virus
EMD: Empirical modal decomposition
BPNN: Back-propagation artificial neural network model
IMF: Intrinsic mode function
SARIMA: Seasonal autoregressive integrated moving average
BP-ANN: Back-propagation neural network
ACF: Autocorrelation function
PACF: Partial autocorrelation function
AIC: Akaike information criterion
SPSS11.5: 11.5 version of Statistical Package for Social Science
